# Yale Brain Atlas to interactively explore multimodal structural and functional neuroimaging data

**DOI:** 10.3389/fnetp.2025.1585019

**Published:** 2025-06-18

**Authors:** Evan Collins, Omar Chishti, Hari McGrath, Sami Obaid, Alex King, Edwin Qiu, Ellie Gabriel, Xilin Shen, Jagriti Arora, Xenophon Papademetris, R. Todd Constable, Dennis D. Spencer, Hitten P. Zaveri

**Affiliations:** ^1^ Department of Neurosurgery, Yale School of Medicine, New Haven, CT, United States; ^2^ Department of Biological Engineering, Massachusetts Institute of Technology, Cambridge, MA, United States; ^3^ David H. Koch Institute for Integrative Cancer Research, Massachusetts Institute of Technology, Cambridge, MA, United States; ^4^ Max Planck School of Cognition, Leipzig, Germany; ^5^ Department of Clinical Neurosciences, University of Cambridge, Cambridge, United Kingdom; ^6^ Division of Neurosurgery, Department of Surgery, Faculty of Medicine, University of Montreal, Montreal, QC, Canada; ^7^ Neurosurgery Service, University of Montreal Hospital Center (CHUM), Montreal, QC, Canada; ^8^ Department of Plant and Microbial Biology, University of California, Berkeley, Berkeley, CA, United States; ^9^ Chobanian & Avedisian School of Medicine, Boston University, Boston, MA, United States; ^10^ Perelman School of Medicine, University of Pennsylvania, Philadelphia, PA, United States; ^11^ Department of Radiology and Biomedical Imaging, Yale School of Medicine, New Haven, CT, United States; ^12^ Department of Biomedical Engineering, Yale University, New Haven, CT, United States; ^13^ Department of Biomedical Informatics and Data Science, Yale School of Medicine, New Haven, CT, United States; ^14^ Interdepartmental Neuroscience Program, Yale University, New Haven, CT, United States; ^15^ Department of Neurology, Yale School of Medicine, New Haven, CT, United States

**Keywords:** brain atlas, web tool, neuroimaging, structure-function, network physiology, connectivity, fMRI

## Abstract

Understanding the relationship between structure and function in the human brain is essential for revealing how brain organization influences cognition, perception, emotion, and behavior. To this end, we introduce an interactive web tool and underlying database for Yale Brain Atlas, a high-resolution anatomical parcellation designed to facilitate precise localization and generalizable analyses of multimodal neuroimaging data. The tool supports parcel-level exploration of structural and functional data through dedicated interactive pages for each modality. For structural data, it incorporates white matter connectomes of 1,065 subjects and cortical thickness profiles of 200 subjects both from the Human Connectome Project. For functional data, it includes resting-state fMRI connectivity matrices for 34 healthy subjects and task-specific fMRI activation data acquired from two meta-analytic resources–Neurosynth and NeuroQuery–which, once translated into Yale Brain Atlas space and modified to include 334 function-specific terms, form Parcelsynth and ParcelQuery, respectively. Altogether, to support investigation of brain structure-function relationships, this study presents a web tool and database for the Yale Brain Atlas that enable scalable, interactive exploration of multimodal neuroimaging data.

## 1 Introduction

The field of neuroscience has evolved to incorporate a variety of methodological approaches for studying brain organization and function, including structural and functional neuroimaging, electrophysiological recordings, computational modeling, and molecular techniques (Loosen et al., 2024; Ng et al., 2024; Rizzolatti et al., 2018). These approaches provide complementary measurements of brain structure, connectivity, and neural activity across different spatial and temporal scales. However, integrating data from these different approaches remains complex. Structural and functional neuroimaging, particularly in the clinical sciences, benefit from consistent spatial frameworks to standardize data analysis and allow comparison across subjects and studies, yet the availability of data tools to support this task remains limited.

For this purpose, in a prior study, we developed Yale Brain Atlas (YBA), a high-resolution anatomical atlas with comprehensive nomenclature well-suited for precise localization of multimodal neuroscience data (McGrath et al., 2022). The focus on facilitating multimodality was built into the design of YBA–the parcels of YBA are approximately 1 cm apart on the cortical surface, equivalent to the distance between intracranial electroencephalography electrode contacts commonly used in epilepsy surgery and neuro-oncology (Berger et al., 1993; Yang et al., 2014; Yao et al., 2017; Zaveri et al., 2009). Our studies have examined the usefulness of YBA in localizing the seizure onset zones for epilepsy research (Sivaraju et al., 2024) and mapping structure-function gradients in systems neuroscience (Collins et al., 2024). In future clinical applications, YBA’s anatomically precise and functionally informed framework could help guide electrode placement for neuromodulation or selection of resection targets that mitigate functional damage. However, despite its demonstrated utility and future potential, YBA has lacked a centralized, interactive platform to explore the atlas and multimodal data.

While a number of brain atlases have been widely adopted for neuroimaging analysis–including anatomical atlases such as Destrieux (Destrieux et al., 2010), Harvard-Oxford (Desikan et al., 2006; Frazier et al., 2005; Makris et al., 2006), and Automated Anatomical Labelling (AAL) (Rolls et al., 2020), as well as functional and connectivity-based parcellations such as Glasser (Glasser et al., 2016), Schaefer (Schaefer et al., 2018), and Brainnetome (Fan et al., 2016) – these resources are generally distributed as static label maps or surface-based files and are not paired with centralized web tools for interactive, multimodal data exploration. Some platforms, such as the Brainnetome viewer (Fan et al., 2016) and the EBRAINS viewer (Amunts et al., 2020), offer some interactivity and include limited modalities such as connectivity profiles or cytoarchitectonic features. Others, like the Allen Brain Atlas viewer (Ding et al., 2016), are focused primarily on molecular and histological data rather than MRI-based neuroimaging. To our knowledge, no existing high-resolution atlas provides an integrated, parcel-level web tool for exploring multiple MRI-derived datasets–such as white matter connectomes, cortical thickness, resting-state fMRI, and meta-analytic task activations–within a unified, anatomically-grounded framework. This accessibility gap motivated us to develop a new interactive platform for the high-resolution YBA with expanded multimodal neuroimaging data.

Here, we introduce a unified web tool and expanded database (https://yalebrainatlas.github.io/YaleBrainAtlas/) that enable interactive exploration of YBA parcels and structural and functional neuroimaging data. The web tool is organized into modality-specific interactive pages, each designed to facilitate parcel-level exploration of the data, as well as dedicated pages for examining the YBA parcellation itself. The underlying database is designed to accommodate future data additions, ensuring scalability as new datasets become available in YBA space. For structural data, the tool incorporates the white matter (WM) connectomes of 1,065 subjects originally sourced from the Human Connectome Project (HCP). It also includes cortical thickness profiles of 200 subjects originally sourced from HCP. For functional data, it includes resting-state functional magnetic resonance imaging (rsfMRI) correlation connectivity matrices for 34 healthy subjects imaged at Yale. It also includes task-specific fMRI activation data acquired from two meta-analytic web-scraped resources–Neurosynth (Yarkoni et al., 2011) and NeuroQuery (Dockès et al., 2020) – which, once translated into YBA space and modified to include 334 function-specific terms, form Parcelsynth and ParcelQuery, respectively. Altogether, this study provides an easy-to-use interactive platform for exploring YBA and its associated multimodal data.

## 2 Methods

### 2.1 Yale brain atlas

As introduced in our prior study (McGrath et al., 2022), YBA is a high-resolution anatomical landmark-based atlas in MNI152 (ICBM 2009a nonlinear symmetric 1 × 1 × 1 mm) space covering the cortex, insula, hippocampus, amygdala, and corpus callosum. The technical details of YBA and neuroimaging data are recorded in Table 1 (see following Methods subsections for more information about each neuroimaging modality). YBA is provided as two versions–one with 696 parcels, and another with 690 parcels which excludes six parcels in the corpus callosum. For some modalities such as cortical thickness, ascribing values to corpus callosum parcels is impractical; thus, this reduced version of YBA is provided. For each YBA version, this study provides the coordinates and indices, parcel dictionary, and three-dimensional mesh files for easy, accessible use.

**TABLE 1:** Technical details of Yale Brain Atlas and its multimodal data organized in this study.

Data type	Data descriptions	Sample size	References
Yale Brain Atlas	For either 690- or 696-parcel atlas versions - Coordinates and indices - Parcel dictionary - 3D mesh	690 or 696 parcels	McGrath et al. (2022)
White matter connectome	- Number of white matter streamlines and white matter length between parcels for each subject - Pairwise structural connectivity between parcels averaged across all subjects for 12 different SC metrics - 3D mesh	1,065 healthy adult subjects from HCP	Yeh (2022) Collins et al. (2024)
Cortical thickness	- Cortical thickness values (mm) between parcels averaged for each subject - Mean cortical thickness values (mm) between parcels averaged across all subjects	200 healthy adult subjects from HCP	Collins et al. (2024)
rsfMRI	- rsfMRI pairwise Pearson correlation coefficients between parcels for each subject - rsfMRI pairwise Pearson correlation coefficients between parcels averaged across all subjects	34 healthy adult subjects imaged at Yale	Collins et al. (2024)
Parcelsynth	- fMRI activation z-score values for 1,334 terms (all terms unmodified from Neurosynth) for each parcel - fMRI activation z-score values for 334 functional terms for each parcel - Publications that report activations for each parcel - Coordinates of reported activations for each parcel	334 functional terms from 14,371 neuroimaging publications	Yarkoni et al. (2011) Collins et al. (2024)
ParcelQuery	- fMRI activation z-score values for 334 functional terms for each parcel	334 functional terms from 13,459 neuroimaging publications	Dockès et al. (2020) Collins et al. (2024)

### 2.2 White matter connectome

The white matter (WM) connectome consists of tracts of axons that make up neural circuits, composing both short- and long-distance interactions between brain regions (Douglas and Martin, 2004; Mišić and Sporns, 2016; Sporns et al., 2005). These neuronal connections are regarded as the physiological basis of behavior, perception, and cognition (Wei et al., 2021). This study provides the processed WM connectomes for 1,065 healthy subjects in YBA space and summary statistics, i.e., pairwise structural connectivity (SC) between parcels averaged across all subjects for 12 different SC metrics described in further detail below.

As introduced in our prior study (Collins et al., 2024), we used WM connectome data from Yeh (Yeh, 2022), which includes tractograms processed from diffusion MRI (dMRI) data from 1,065 healthy young adult subjects (575 females, 490 males; mean age 28.74 years; age range from 22 to 37 years). Yeh sourced the dMRI data from the Human Connectome Project (HCP), converted it to the DSI Studio file format, and reconstructed it in MNI common space using q-space diffeomorphic reconstruction (Yeh, 2022; Yeh and Tseng, 2011). Details regarding the deterministic fiber tracking algorithm used can be found in our prior study (Collins et al., 2024).

A total of one million streamlines were created for each subject. Two iterations of topology-informed pruning were executed to remove false positive streamlines. To generate a SC matrix for WM streamline count for each subject, we computed the number of streamlines with endpoints in distinct parcel pairs. Next, to generate a representative group-level SC matrix for WM streamline count (SC-Count), we averaged the subject-level WM streamline count SC matrices. Following a prior study (Zamani Esfahlani et al., 2022), the group-level SC-Count matrix was normalized by dividing by the geometric mean volume of the two parcels. Although the YBA parcels are all roughly the same size at the cortical surface, they have significant differences in volumes (mean: 921.94 mm^3^; SD: 518.27 mm^3^). This volume-normalization step is essential for being able to compare between parcels without the streamline counts being skewed by differences in parcel volume. We did not apply zeroing thresholds or length-based weighting to this volume-normalized group-level SC-Count matrix.

For each of the 1,065 subjects, we also computed the SC matrix for WM length (SC-Length) as the arithmetic mean of streamline lengths between any connected parcels. A representative group-level SC-Length matrix was computed as the average among the subject-level matrices. Next, we computed the SC matrix for Euclidean distance (SC-Eucl Dist) by taking the Euclidean distance between parcel centroids. These group-level matrices of SC-Count, SC-Length, and SC-Eucl Dist were subsequently inputted into network analysis scripts from Zamani Esfahlani et al. (2022) to generate additional group-level SC metrics. These additional SC matrices are computed from seven different SC metrics: cosine similarity (SC-Cosine), flow graphs (SC-Flow Graph), matching index (SC-Matching Index), navigation count (SC-Nav Count), navigation length (SC-Nav Length), search information (SC-Search Info), and mean first passage time (SC-MFPT). We computed two additional matrices by two more SC metrics: path length (SC-Path Length) and path count (SC-Path Count). Mathematical definitions of each metric can be found in the prior studies (Collins et al., 2024; Zamani Esfahlani et al., 2022). In total, this dataset thus includes 12 different group-level SC metrics. The web tool allows users to interactively examine SC among the YBA parcels.

### 2.3 Cortical thickness

From the 1,065 healthy young adult subjects from HCP referenced for WM connectomes, we selected a representative subset of 200 subjects (100 females, 100 males; mean age 28.85 years; age range from 23 to 36 years) for the cortical thickness data provided in this study. We processed non-skull stripped T1-weighted images for the 200 subjects using Advanced Normalization Tools (ANTs) software to generate cortical thickness values for gray matter. The results were nonlinearly normalized to MNI152 space. The cortical thickness values of the voxels making up each of the 690 YBA parcels (6 corpus callosum parcels excluded) were averaged to produce a representative cortical thickness value per parcel. Using the cortical thickness values per parcel for the 200 subjects, we also computed an averaged cortical thickness map. The web tool allows users to interactively examine the cortical thickness of each YBA parcel.

### 2.4 rsfMRI

This study provides rsfMRI pairwise correlation connectivity matrices for 34 healthy adult subjects. As introduced in our prior study (Collins et al., 2024), fMRI imaging for each subject was performed on a 3-T Siemens Trio scanner (Siemens Medical Systems, Erlangen, Germany) using a 64-channel head coil for the 34 subjects (17 females, 17 males; mean age 33 years; age range 18–55 years) (IRB 1003006485 & 0,702,002,395). Informed consent was signed by all subjects. Each subject was positioned within the coil, and head movements were reduced with added pillows. Additional technical details regarding the rsfMRI study can be found in our prior study (Collins et al., 2024).

To align the individual subject data to a common reference space, we performed sequential registrations within Yale BioImage Suite (Papademetris et al., 2006) in two steps: (1) a linear registration between the individual subject’s rsfMRI image and their anatomical image, and (2) a nonlinear registration between the subject’s anatomical image and the standard whole-brain template (MNI152 1 mm). Mean rsfMRI activations across the entire time course were computed for each parcel. Finally, the connectivity matrix was obtained using the Fisher transformation of the Pearson correlation coefficient for each pair of parcels. Hence, the final output from this processing was a rsfMRI pairwise correlation connectivity matrix (i.e., FC-rsfMRI) of size 696 by 696 (parcels) for each of the 34 healthy subjects. An averaged group-level rsfMRI connectivity matrix was also computed. The web tool allows users to interactively examine FC-rsfMRI among the YBA parcels.

### 2.5 Parcelsynth

This study provides the dataset for Parcelsynth, which was introduced in our prior study (Collins et al., 2024). Parcelsynth is the fMRI activation database of Neurosynth (Yarkoni et al., 2011) translated into YBA space for 334 function-specific terms. Neurosynth has aggregated fMRI activation data from 14,371 studies, recording voxelwise z-score maps of activations across 1,334 terms. The z-scores by term in Neurosynth were computed from a one-way ANOVA evaluating whether the proportion of studies that record activation at a specific voxel differs from the proportion that would be expected if activations were uniformly distributed in gray matter. The 1,334 terms originate from topic analysis of the web-scraped studies, which include both resting-state and diverse task-based studies. The number of terms to include in Neurosynth (i.e., 1,334) was determined by a minimum threshold for the number of studies involving each term (Yarkoni et al., 2011). As described in our prior study (Collins et al., 2024), we reduced this list to 334 terms by (1) removing the terms describing locations (“prefrontal”) and (2) combining redundant terms (e.g., “emotional” and “emotion”). The resulting list of 334 terms reflects diverse function-specific terms (e.g., “write”). For terms that we combined, we averaged their activation data in Neurosynth. Next, to translate the Neurosynth data into YBA space, we averaged activation z-scores from the voxels contained within each YBA parcel for each of the 334 functional terms, resulting in Parcelsynth, i.e., the averaged z-scores associated with 334 functional terms for 696 YBA parcels. Our dataset also provides a functional connectivity matrix with dimensions 696 by 696 (parcels) based on the Parcelsynth data by computing the cosine similarity between parcel pairs. The web tool allows users to interactively examine different summary statistics of Parcelsynth activation data by specific YBA parcel or by specific function.

### 2.6 ParcelQuery

This study provides the dataset for ParcelQuery, which was also first detailed in our prior study (Collins et al., 2024). ParcelQuery is the fMRI activation database of NeuroQuery (Dockès et al., 2020) translated into YBA space for 334 function-specific terms. Similar to Neurosynth, NeuroQuery is a meta-analytic resource for fMRI activation data across a variety of terms. NeuroQuery expands on Neurosynth primarily through a larger, updated corpus of text (i.e., approximately 75 million words) and the use of semantic smoothing based on word embeddings to synthesize activation maps (Dockès et al., 2020). We analyzed the fMRI activation data from NeuroQuery using the same set of 334 functional terms previously selected from Neurosynth. Similar to our approach with Neurosynth in forming Parcelsynth, we computed the average NeuroQuery activation z-scores for each of the 334 functional terms across voxels contained within each YBA parcel, resulting in ParcelQuery. Our dataset also provides a functional connectivity matrix with dimensions 696 by 696 (parcels) based on this ParcelQuery data by computing the cosine similarity between parcel pairs. The web tool allows users to interactively examine different summary statistics of ParcelQuery activation data by specific YBA parcel or by specific function.

## 3 Results

**FIGURE 1:**
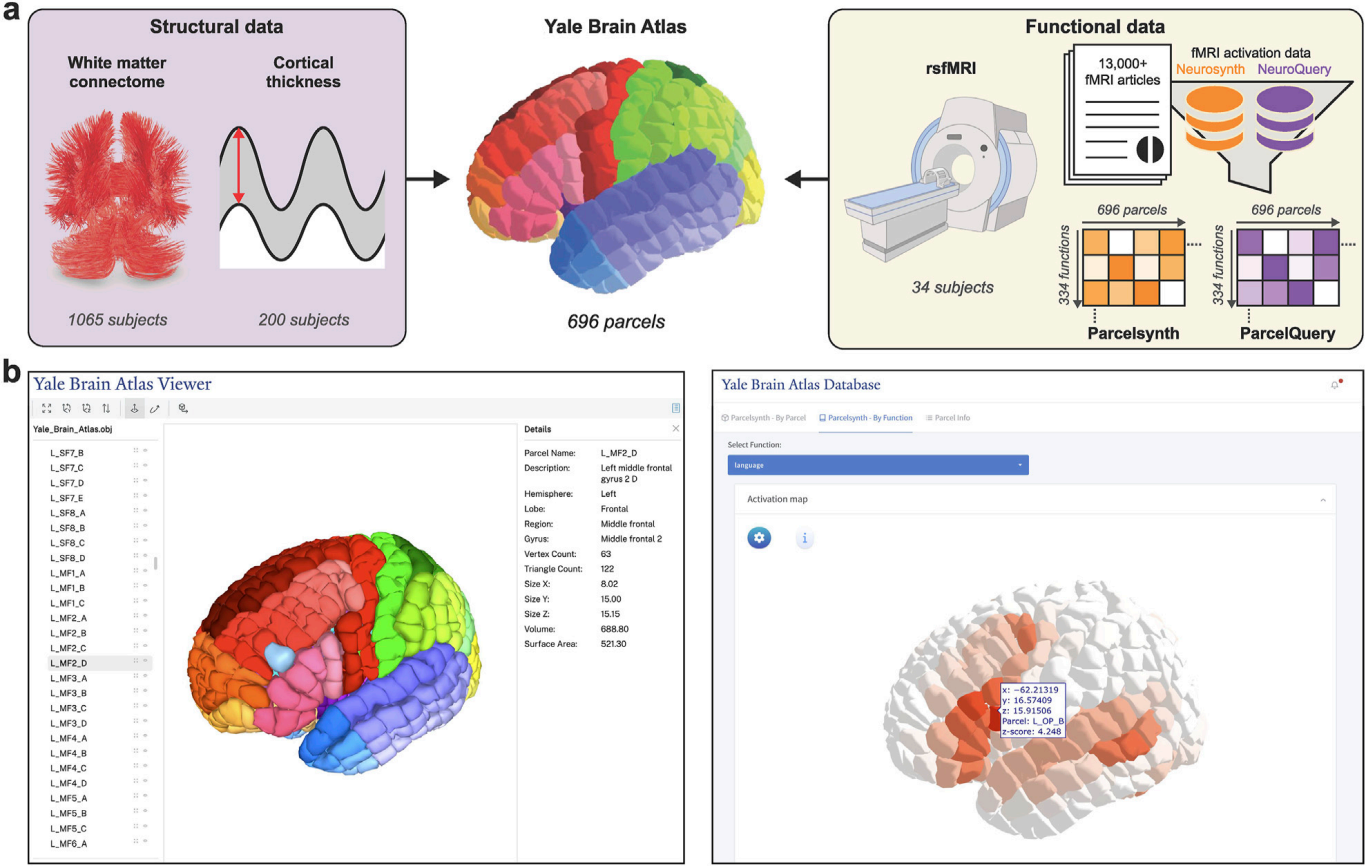
Localizing and interactively viewing multimodal data in Yale Brain Atlas space. **(a)** Multimodal structural and functional neuroimaging data are acquired and translated into the 696 parcels of Yale Brain Atlas (YBA) space, a high-resolution anatomical parcellation well suited for precise yet generalizable analyses. Structural data translated into YBA space includes white matter tractographies processed from 1,065 healthy subjects from the Human Connectome Project (HCP) and cortical thickness data processed from 200 healthy subjects from HCP. Functional data includes resting-state fMRI (rsfMRI) correlation connectivity matrices for 34 healthy subjects imaged at Yale and task-specific fMRI activation data acquired from two meta-analytic resources–Neurosynth and NeuroQuery–which, once translated into YBA space and modified to only include specific functional terms (e.g., “write”), form Parcelsynth and ParcelQuery, respectively. **(b)** The atlas and its multimodal data can be interactively viewed at https://yalebrainatlas.github.io/YaleBrainAtlas/. Interactive viewer for the parcels themselves is shown left. Interactive viewer for “language” fMRI activation in Parcelsynth is shown right.

In this study, we introduce interactive web tool and accompanying structural and functional neuroimaging data for YBA. With relatively small and uniform parcel size (i.e., approximately 1 cm^2^ on cortical surface) coupled with standardized parcel nomenclature, YBA supports precise yet generalizable analyses, as demonstrated in our prior study on structure-function gradients (Collins et al., 2024). Altogether, YBA provides a precise anatomical and functional reference framework for scientific and clinical applications. Motivated by the lack of publicly accessible tools for exploring multimodal datasets in standardized atlas space, this study centralizes all current YBA resources and multimodal neuroimaging data into a single downloadable resource and accompanying interactive web tool (Figure 1).

**FIGURE 2:**
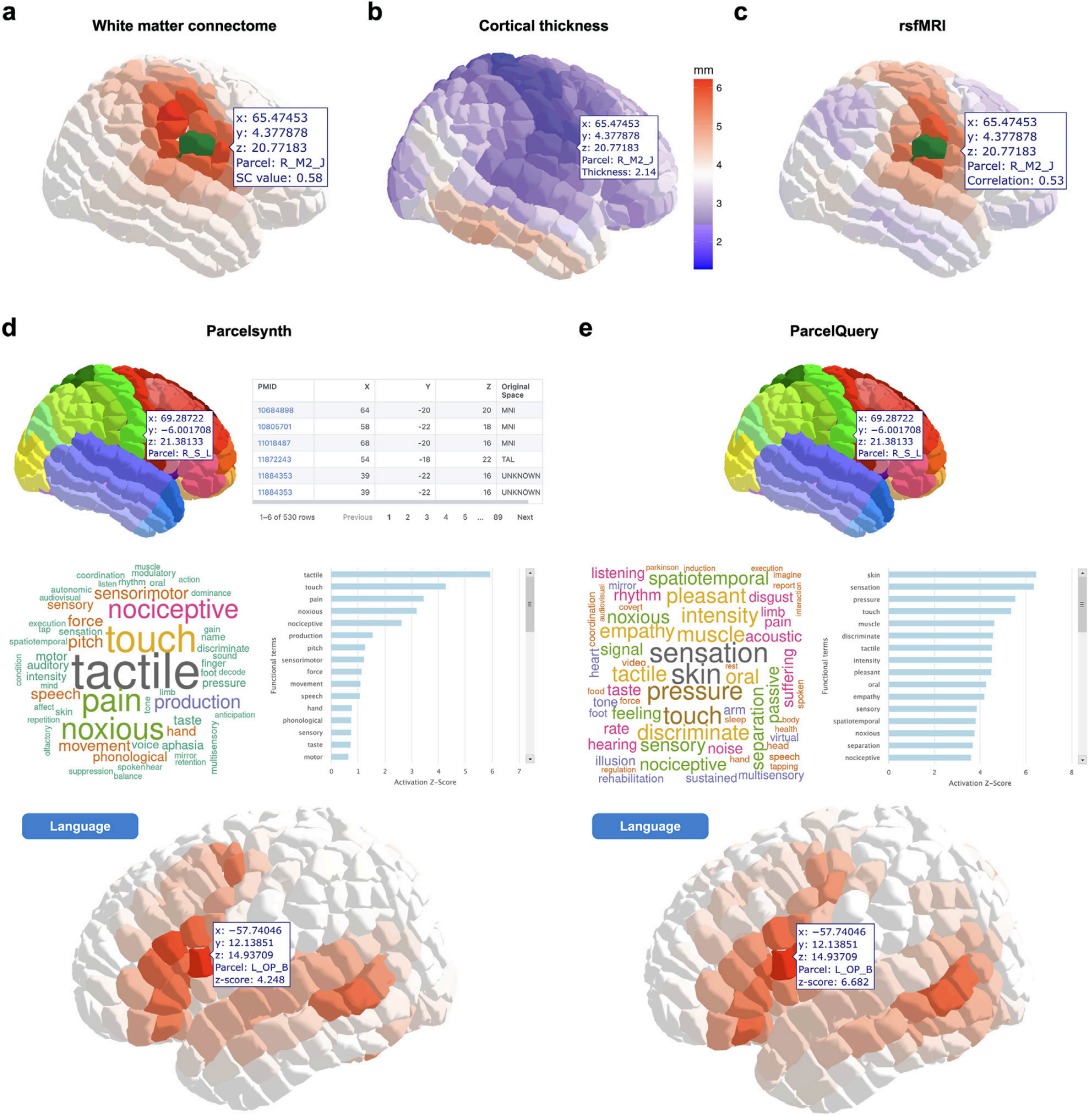
Example interactive views of neuroimaging data in Yale Brain Atlas space. **(a)** Example view of white matter connectome data in Yale Brain Atlas (YBA) space. In this case, the structural connectivity (SC) metric “cosine” and the parcel “R_S_L” (shown in green) have been selected. The other 695 parcels are shaded according to their cosine SC value with respect to the selected parcel R_S_L. The user hovers the cursor over another parcel, R_M2_J, and an overlay displays its SC value with R_S_L. **(b)** Example view of cortical thickness data in YBA space. The parcels are shaded according to cortical thickness. In this case, the user hovers the cursor over a parcel, R_M2_J, to see its thickness value. **(c)** Example view of resting-state fMRI (rsfMRI) connectivity data. In this case, the parcel R_S_L (shown in green) has been selected. The user hovers the cursor over another parcel, R_M2_J, to see its Pearson correlation coefficient value with respect to the selected parcel R_S_L. **(d)** Example view of Parcelsynth. In this case, the parcel R_S_L has been selected (top left). Tables of the papers and coordinates reporting activations in that selected parcel are shown (top right). The specific functional terms with the greatest activation z-scores for the selected parcel R_S_L are shown in a wordcloud figure (middle left) and barplot figure (middle right). Instead of selecting a specific parcel, the user can select a specific functional term, such as “language”. The user can then hover the cursor over each parcel, e.g., L_OP_B, to see its activation z-score for the selected functional term “language” (bottom). **(e)** Analogous to **(d)** for ParcelQuery. Example views shown; to explore more features and visualizations, visit https://yalebrainatlas.github.io/YaleBrainAtlas/.

The website includes pertinent references and links to download each of the featured datasets. In addition to a parcel viewer, the web tool features interactive viewers for each of the five neuroimaging modalities described in Methods. These include white matter connectomes, cortical thickness, rsfMRI, and meta-analytic Parcelsynth and ParcelQuery. The tool is designed to be modular and expandable, supporting future data additions (Figure 2).

## 4 Discussion

Yale Brain Atlas (YBA) has been designed as a high-resolution anatomical atlas to facilitate the integration of multimodal neuroscience data with precise spatial localization. This study builds on the YBA framework by developing a centralized, interactive web tool and expanding the underlying structure-function neuroimaging data mapped in YBA space, including white matter connectomes, cortical thickness profiles, resting-state fMRI connectivity, and meta-analytic task-based fMRI activations. By mapping all datasets to a high-resolution anatomical framework with standardized nomenclature, this platform facilitates reproducible and generalizable analyses across domains of neuroscience and clinical research. Compared to existing atlases that offer static label maps or modality-specific resources, this tool fills an important accessibility gap by integrating multiple MRI-derived modalities within a single, user-friendly web environment. We anticipate that this tool will be useful for hypothesis generation, region-of-interest selection, cross-modal comparison, future data compilation, and educational applications.

The structure-function datasets and associated summary statistics mapped to YBA space in this study contribute to its role as a reference framework for studying brain organization. Moreover, the platform allows for the addition of datasets, supporting future extensions of the resource. Analyses conducted in YBA space can continue to examine typical network organization and identify changes associated with neurological disorders such as epilepsy (Collins et al., 2024; Sivaraju et al., 2024) and, in the future, could help guide electrode localization or resection strategies. However, the current implementation has limitations to motivate future research. The atlas is defined in a common reference space (MNI152), which may reduce its applicability in studies requiring subject-level analysis. Additionally, the task-based functional data are sourced from meta-analytic repositories rather than individual-level experimental datasets, which may limit interpretability in some contexts. Future work could consider incorporating other modalities, including molecular or electrophysiological data. Overall, the resources for YBA provided in this study offer a spatial framework for exploring structural and functional relationships across multiple neuroimaging modalities.

## Data Availability

The datasets presented in this study can be found in online repositories. The names of the repository/repositories and accession number(s) can be found below: YBA and its multimodal data can be downloaded either from our GitHub repository https://github.com/YaleBrainAtlas/YaleBrainAtlas/tree/master/data or our NITRC page https://www.nitrc.org/projects/yale_atlas_2021/. All code for the website and accompanying Shiny apps is available on our GitHub repository: https://github.com/YaleBrainAtlas/YaleBrainAtlas.
